# Isolation and identification of *Mycoplasma hyorhinis* and virulence evaluation of its field isolates

**DOI:** 10.3389/fvets.2025.1542992

**Published:** 2025-06-18

**Authors:** Fan Yang, Lijun Yang, Xuecheng Duan, Yulin Qian, Huifang Ma, Xue Jia, Xinyu Huo, Wenqi Dong, Huanchun Chen, Chen Tan

**Affiliations:** ^1^State Key Laboratory of Agricultural Microbiology, College of Veterinary Medicine, Huazhong Agricultural University, Wuhan, China; ^2^Key Laboratory of Preventive Veterinary Medicine in Hubei Province, The Cooperative Innovation Center for Sustainable Pig Production, Wuhan, China; ^3^International Research Center for Animal Disease, Ministry of Science and Technology of the People’s Republic of China, Wuhan, China

**Keywords:** *Mycoplasma hyorhinis*, positivity rate, isolation, identification, infection

## Abstract

**Introduction:**

As a prevalent swine pathogen worldwide, *Mycoplasma hyorhinis* (*M. hyorhinis*, Mhr) is associated with various diseases, including multiple serositis, pneumonia, arthritis, and otitis media. It is also linked to the porcine respiratory disease complex (PRDC).

**Methods:**

*M. hyorhinis* prevalence in 2022 Chinese lung samples was assessed by species-specific PCR, followed by isolation and purification of field strains, followed by genetic characterization via multilocus sequence typing (MLST). Pathogenicity evaluation of three isolates (ZZ-1, GD-1 and AH-1) was evaluated using controlled piglet infection trials.

**Results:**

Mhr detection in clinical lung samples showed 31.77% prevalence. Three isolates (ZZ-1/ST166, GD-1/ST167, AH-1/ST144) were characterized by MLST. Piglet infection trials confirmed Mhr-induced polyserositis, pneumonia, and arthritis, with strain-dependent virulence variation observed.

**Discussion:**

This study confirms *M. hyorhinis* as a high-prevalence pathogen (31.77%) in Chinese swine herds. Animal infection models demonstrated virulence variation among different Mhr strains. These findings contribute to identifying and assessing the threats posed by different strains to pig health, guiding the development of clinical prevention and control strategies.

## 1 Introduction

*Mycoplasma hyorhinis* (*M. hyorhinis*, Mhr) is a globally prevalent swine pathogen that has been largely understudied. While it is a common respiratory bacterium, most pigs colonized with Mhr exhibit no apparent clinical symptoms (1, 2). However, Mhr can cause various diseases, including swine multiple serositis, pneumonia, pericarditis, pleuritis, arthritis, conjunctivitis, and otitis media (3–11). Recent studies have shown that Mhr is also associated with meningitis and endocarditis (12–14). It is also recognized as a contributing pathogen in the porcine respiratory disease complex (PRDC) (15, 16). The prevalence of Mhr varies geographically and between herds. In this respect, in Switzerland, 10% of lung tissues from pigs with pneumonia tested positive for Mhr, while in the United States, PCR testing revealed that up to 98% of weaned piglets were positive (17). Mhr can cause enzootic pneumonia (EP) independently, although *Mycoplasma hyopneumoniae* is the primary pathogen associated with EP (4, 18–21). Mhr colonizes the ciliated epithelium of the respiratory tract and can typically be isolated from nasal secretions, tonsils, or bronchoalveolar lavage fluid (BALF) (9, 22, 23). Some researchers have also isolated Mhr from lung tissue (4, 24).

Clinical symptoms typically manifest in piglets between 3 and 10 weeks of age, although older pigs can occasionally be affected (25). In younger piglets, infection can lead to emaciation and reduced daily weight gain (26). Infected pigs may exhibit lethargy, decreased appetite, lameness, and dyspnea (5, 22). The primary anatomical and pathological findings include polyserositis and arthritis, with occasional occurrences of pneumonia (4, 27, 28).

It has been hypothesized that systemic dissemination plays a crucial role in *M. hyorhinis*-associated diseases (29–31). However, the precise mechanisms underlying the systemic dissemination of Mhr, leading to conditions such as polyserositis and arthritis, remain poorly understood. Some researchers propose that co-infection with other pathogens and stress factors may promote the systemic spread of Mhr (6, 31). Several *in vivo* infection studies involving various field isolates of Mhr have been conducted (4, 22), indicating that strains with different virulence levels exhibit varying pathogenic potentials.

To investigate the prevalence of Mhr in clinical lung tissue in China, 1,001 lung tissue samples were collected from 25 provinces in 2022 and analyzed using a specific qPCR assay targeting the *p37* gene. Three Mhr strains were successfully isolated and purified from these samples. An Mhr infection model was established in piglets through multiple routes of infection to assess the virulence of different strains and to investigate whether all Mhr strains isolated from lung tissue exhibit pathogenicity. This study also provides a foundation for further research into the pathogenic mechanisms of Mhr.

## 2 Materials and methods

### 2.1 Identification of *Mycoplasma hyorhinis* in pig lung samples

Clinical samples randomly collected from pig farms across various regions of China in 2022 were processed for DNA extraction using an Automatic total DNA extraction machine (Vazyme, China) and analyzed using specific qPCR to detect Mhr, as described in a previous study (32). Mhr presence in a sample was confirmed when the qPCR results targeting the *P37* gene showed a Ct value of ≤35. The primer and probe sequences were as follows: Forward primer 5′-AGAAGGTTCTTTTGCTTGAACACA-3′, Reverse primer 5′-TGCTTCCATCTTTTCATTTGCTT-3′, and Probe 5′-FAM-ATCAGCAACAAAACCTT-MGB-3′. The PCR master mix consisted of 10 μL 2 × *Taq* Master Mix (Vazyme, China), 0.8 μL of primer, 0.2 μL of probe, and 9 μL of DNA. PCR conditions were as follows: 37°C for 2 min, 95°C for 5 min, and 45 cycles of 95°C for 10 s and 55.9°C for 30 s.

### 2.2 Isolation and purification of *Mycoplasma hyorhinis*

#### 2.2.1 Methods for the isolation and purification of *Mycoplasma hyorhinis*

Sampling was performed at the lesion margins of fresh lung tissue to optimize Mhr isolation. Approximately 2.0 grams of each sample was collected, finely minced in a biosafety cabinet, and placed in a 2.0 mL centrifuge tube with an equal volume of PBS buffer. After grinding at 60 Hz for 5 min, the homogenate was centrifuged at 3000 rpm for 3 min. The supernatant was filtered through a 0.45 μm filter, transferred to Friis medium, and incubated at 37°C. Once the broth culture changed color, 100 μL of the culture was spread evenly onto a solid medium and incubated at 37°C. Individual colonies were picked and purified by inoculating them into liquid broth. Pure cultures were obtained after three rounds of purification on solid plates.

The isolates were identified using regular PCR targeting the *P37* gene and 16S rRNA with *Mycoplasma* genus-specific primers. The primer sequences were as follows: P37 forward primer 5′-TTGCTCAAAAAATTTAAAAATTT-3′, P37 reverse primer 5′-AACAAAAATTTTATTAATTTCTTTA-3′; 16S rRNA forward primer 5′-GATGAACGCTCGCTGTGTGCCTA-3′, 16S rRNA reverse primer 5′-CTTCACCCCTGTCATCAGTCCT-3′. The PCR master mix consisted of 12.5 μL 2 × Rapid Taq Master Mix (Vazyme, China), 2 μL of each primer, 2 μL of DNA template, and 8.5 μL of ddH_2_O. PCR conditions were as follows: initial denaturation at 95°C for 3 min, followed by 30 cycles of denaturation at 95°C for 15 s, annealing at 52°C for 15 s, and extension at 72°C for 15 s, with a final extension at 72°C for 5 min.

#### 2.2.2 Multilocus sequence typing (MLST) analysis

To determine the genotypes of Mhr isolates in this study, a PCR-based MLST analysis was employed. Primers for six housekeeping genes (*adk, gmk, gltX, rpoB, dnaA, gyrB*) were synthesized following established protocols, referencing Jolley et al. (33). Following MLST analysis, the housekeeping gene sequences were uploaded to the same website to obtain allele values and assign sequence types (STs) for the six genes (34). A list of oligonucleotide primers used to amplify and sequence Mhr MLST can be found in Additional File 1.

### 2.3 Animal experiment

#### 2.3.1 Bacterial strain preparation and animal grouping

To explore the diversity in virulence among different *M. hyorhinis* strains, three field isolates with high and stable growth titers were selected for virulence evaluation. These isolates were obtained from the lung tissues of pigs in Hunan (ZZ-1 strains), Guangdong (GD-1 strains), and Anhui (AH-1 strains) provinces in China, with titers reaching 10^10^ to 10^11^ CCU/mL.

Twelve four-week-old hybrid piglets, free of Mhr and *M. hyopneumoniae* (Mhp) infection, were randomly divided into four groups: three infection groups (*n* = 3 each) and one negative control group (*n* = 3). Pigs in the infection groups were inoculated with one of three Mhr strains, while the control group received Friis medium. To ensure fresh infection material, purified Mhr isolates were prepared 24 h before inoculation. The medium was used directly for inoculation once its color turned orange-yellow, and the viable cell count of Mhr was determined using the color-changing unit (CCU) method (26).

#### 2.3.2 Infection procedure

A simultaneous inoculation strategy through multiple routes was employed for 4-week-old piglets. Each pig in the infection groups received a total of 10 mL of Mhr broth (4 mL intrapulmonary, 4 mL intraperitoneal, and 2 mL intranasal), with the viable cell count determined on the inoculation day. The negative-control group received the same volume of Friis medium administered via the same routes. Twenty-eight days post-infection, all pigs were euthanized and necropsied.

#### 2.3.3 Clinical symptom observation

Daily clinical observations were conducted from 3 days before inoculation (−3 dpi) to 28 days post-infection (28 dpi) to monitor signs of abnormal respirations, cough, and lameness. A clinical scoring system, adapted from the literature (26) (Additional File 2), was used to assess clinical severity. Body temperatures and weights were monitored throughout the observation period to assess the pigs’ overall health and response to infection.

#### 2.3.4 Histopathological observation

At 28 dpi, all pigs were euthanized via intramuscular injection of Xylazine Hydrochloride (0.2 mg/kg) followed by exsanguination until cardiac arrest. Limb joints were examined for signs of arthritis, while abdominal and thoracic cavities, including the pericardium, were inspected for serositis. Lung tissues were assessed for lesions, and lesion severity was scored based on criteria established in previous studies (22, 26, 35), as detailed in Additional File 3. Lung tissue samples with lobulated or patchy consolidation were collected for tissue fixation, sectioning, and hematoxylin–eosin (HE) staining. Pathological histological changes were observed microscopically.

Tissue samples, including myocardium, pericardial membrane, pericardial fluid, pleural effusion, lung, hilar lymph nodes, tonsils, abdominal effusion, and joint fluid, were tested for Mhr using PCR targeting the P37 gene (550 bp). Mhr was re-isolated from positive tissue samples, and the isolates were subjected to MLST typing to confirm strain identity.

The experiment was approved by the Animal Experimental Ethical Inspection of the Laboratory Animal Centre, Huazhong Agriculture University, under reference number HZAUSW-2024-0079.

### 2.4 Statistical analysis

To compare the virulence of three Mhr isolates, statistical analysis was performed on body temperature, average daily weight gain (ADWG), clinical observation scores (abnormal respirations, cough, lameness, and summary score), and postmortem observation scores (peritonitis, pleuritis, pericarditis, arthritis, pneumonia, and summary score). Data were presented as the mean ± standard deviation (SD). ADWG data were analyzed using a one-way analysis of variance (ANOVA) with effect size analysis for selected pairs. Statistical significance was set at *p* < 0.05.

## 3 Results

### 3.1 The positivity rate of *Mycoplasma hyorhinis* in clinical lung samples

**Figure 1:**
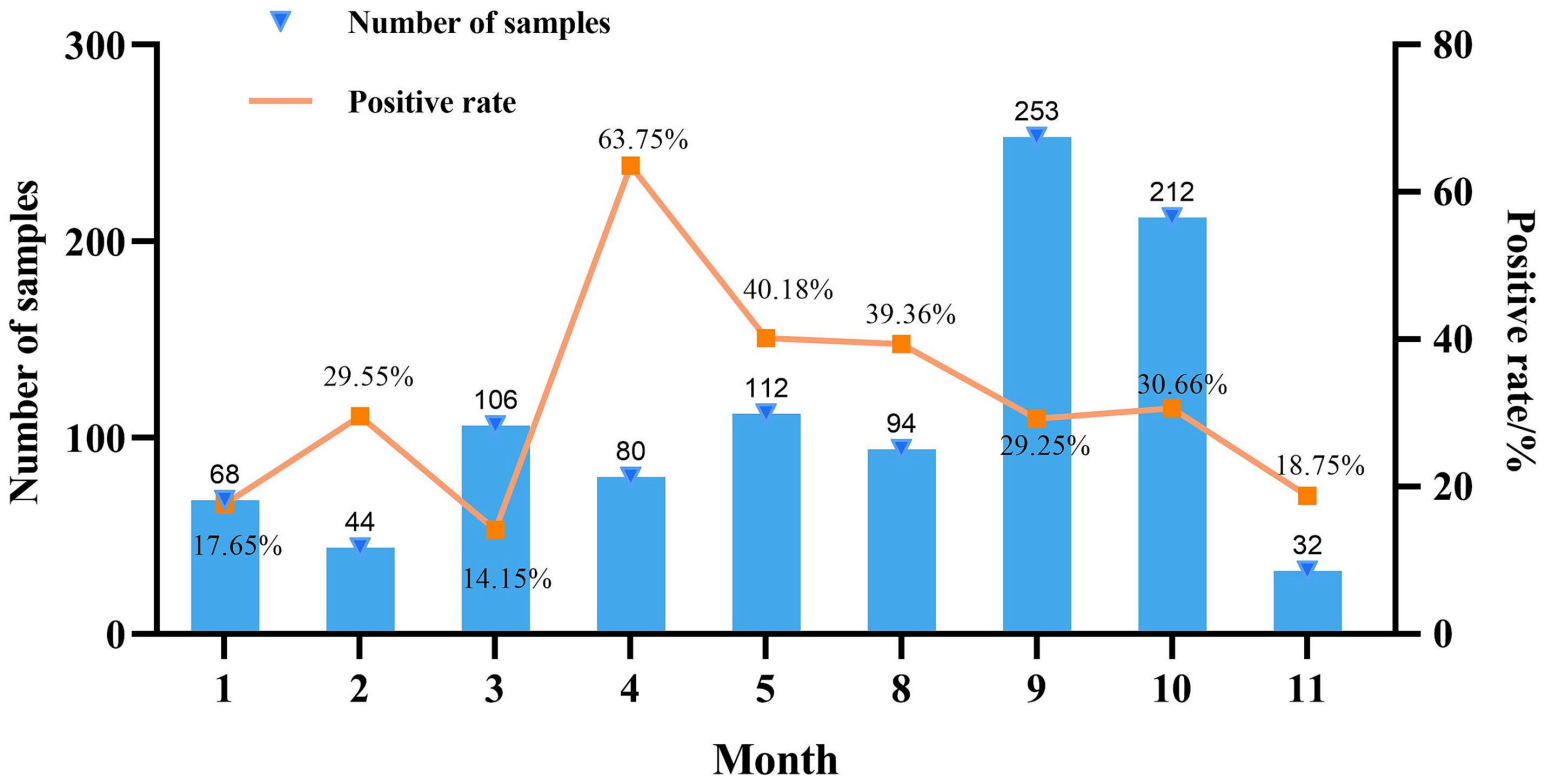
Month-based summary of data collection.

*Mycoplasma hyorhinis*-specific qPCR was performed on 1,001 clinical lung tissue samples collected from 25 provinces in China during 2022. Among these, 13.1% were from Sichuan, 11.4% from Guangdong, 9.8% from Henan, 7.9% from Guangxi, 7.7% from Shaanxi, and 5.1% from Shandong, with the remaining 45.1% originating from 19 other provinces. Among provinces with over 30 samples, Jiangsu exhibited the highest positivity rate for Mhr (40.00%), followed by Hubei (36.73%). Conversely, Anhui and Chongqing had the lowest positivity rates (12.90 and 18.18%, respectively) (Table 1). The highest number of samples were collected in September (253) and October (212), while the fewest were collected in November (32). The positivity rate of Mhr peaked in April (63.75%), May (40.18%), and August (39.36%), and was lowest in January (17.65%), February (29.55%), March (14.15%), and November (18.75%) (Figure 1).

**Table 1:** Province-based summary of data collection.

Province	No. of samples	Proportion of samples	No. of positive samples	Proportion of positive samples
Sichuan	131	13.09%	31	26.72%
Guangdong	114	11.39%	41	35.96%
Henan	98	9.79%	31	31.63%
Guangxi	79	7.89%	29	36.71%
Shaanxi	77	7.69%	24	31.17%
Shandong	51	5.09%	16	31.37%
Hubei	49	4.90%	18	36.73%
Shanxi	47	4.70%	12	25.53%
Hunan	39	3.90%	13	33.33%
Jiangxi	38	3.80%	12	31.58%
Jiangsu	35	3.50%	14	40.00%
Chongqing	33	3.30%	6	18.18%
Anhui	31	3.10%	4	12.90%
Guizhou	30	3.00%	9	30.00%
Hebei	27	2.70%	9	33.33%
Liaoning	26	2.60%	9	34.62%
Fujian	21	2.10%	15	71.43%
Yunnan	20	2.00%	5	25.00%
Zhejiang	13	1.30%	3	23.08%
Inner Mongoria lM	11	1.10%	4	36.36%
Gansu	11	1.10%	5	45.45%
Xinjiang	8	0.80%	1	12.50%
Heilongjiang	7	0.70%	1	14.29%
Tianjin	4	0.40%	2	50.00%
Jilin	1	0.10%	1	100.00%
Total	1001	100%	318	31.77%

### 3.2 Isolation, purification, and sequence typing (ST) of *Mycoplasma hyorhinis*

**Figure 2:**
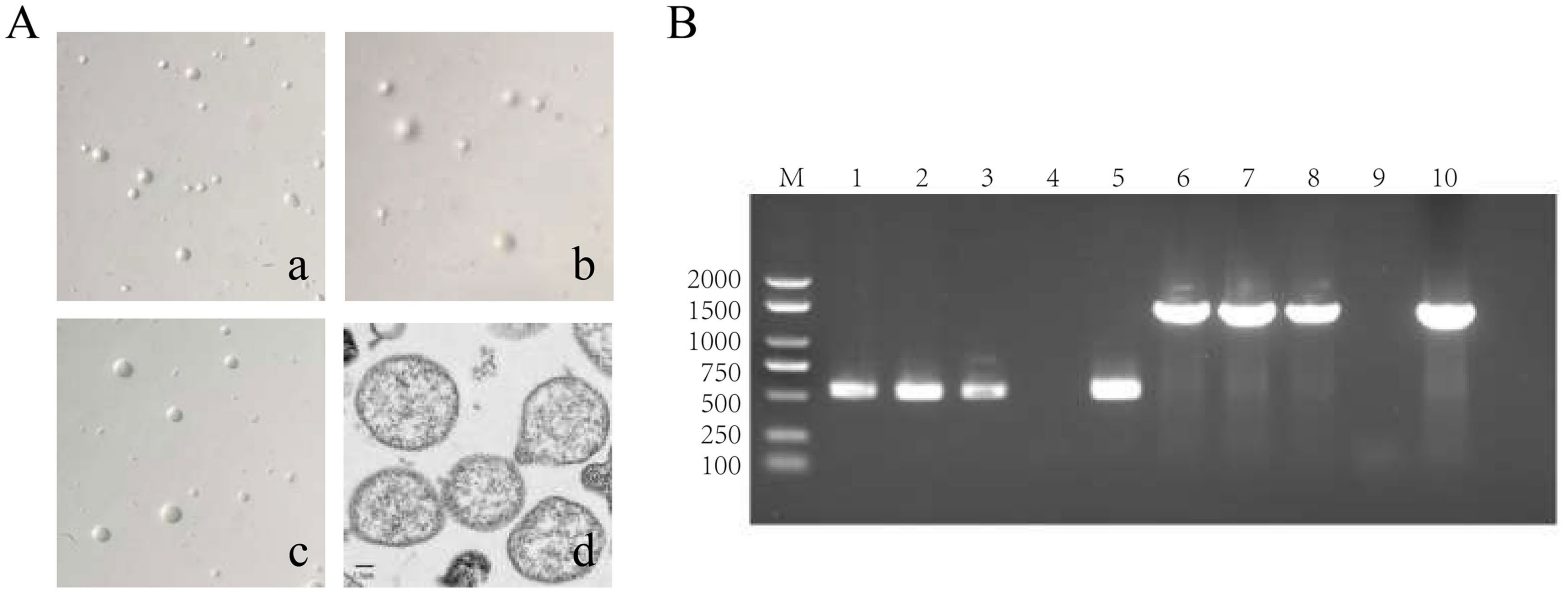
Identification of *M. hyorhinis* isolates. **(A)** Colony morphology of the three Mhr strains on solid media (40×), exhibiting similar “fried egg” colonies. **(a)** ZZ-1 strains; **(b)** GD-1 strains; **(c)** AH-1 strains; **(d)** Transmission electron microscopy (TEM) image of Mhr ZZ-1 strain (Scale bar: 1 μm). The image reveals the typical pleomorphic structure of the Mycoplasma genus, with the cell wall-lacking structures with a granular interior. **(B)** PCR identification of Mhr targeting the P37 gene (550 bp) and 16S rRNA gene (1450 bp). M, DNA marker; lane 1–3, ZZ-1 strain, GD-1 strains and AH-1 strains’ P37 gene amplification; lane 4, negative control; lane 5, positive control (P37 gene); lane 6–8, ZZ-1 strain, GD-1 strains and AH-1 strains’ 16S rRNA gene amplification; lane 9, negative control; lane 10, positive control (16S rRNA gene).

Three strains of *M. hyorhinis* were isolated and purified: ZZ-1, GD-1, and AH-1. After 12–20 h of incubation, the isolates turned the liquid media from red to yellow without visible turbidity. The titers of the three strains reached 1 × 10^9^ to 1 × 10^11^ CCU/ml. On solid media, all strains formed characteristic “fried egg” colonies (Figure 2A). PCR amplification confirmed the presence of the Mhr-specific P37 gene (550 bp) and 16S rRNA gene (1,450 bp) in all three strains (Figure 2B). No exact matches were found for all loci of the ZZ-1 strain in the PubMLST database. New sequences were submitted to the database, and new allele profiles were created, assigning novel ST numbers to their respective allele combinations. Ultimately, the three isolated strains were identified as ST 166 (ZZ-1), ST 167 (GD-1), and ST 144 (AH-1), respectively, which were used in subsequent experiments (Table 2).

**Table 2:** Genotyping of the Mhr strains by MLST.

Strains	Gene alleles						ST
	dnaA	rpoB	gyrB	gltX	adk	gmk	
ZZ-1	26	1	4	4	2	5	166
GD-1	10	25	15	4	15	3	164
AH-1	24	1	1	4	2	5	144

### 3.3 Clinical symptom observations

**Figure 3:**
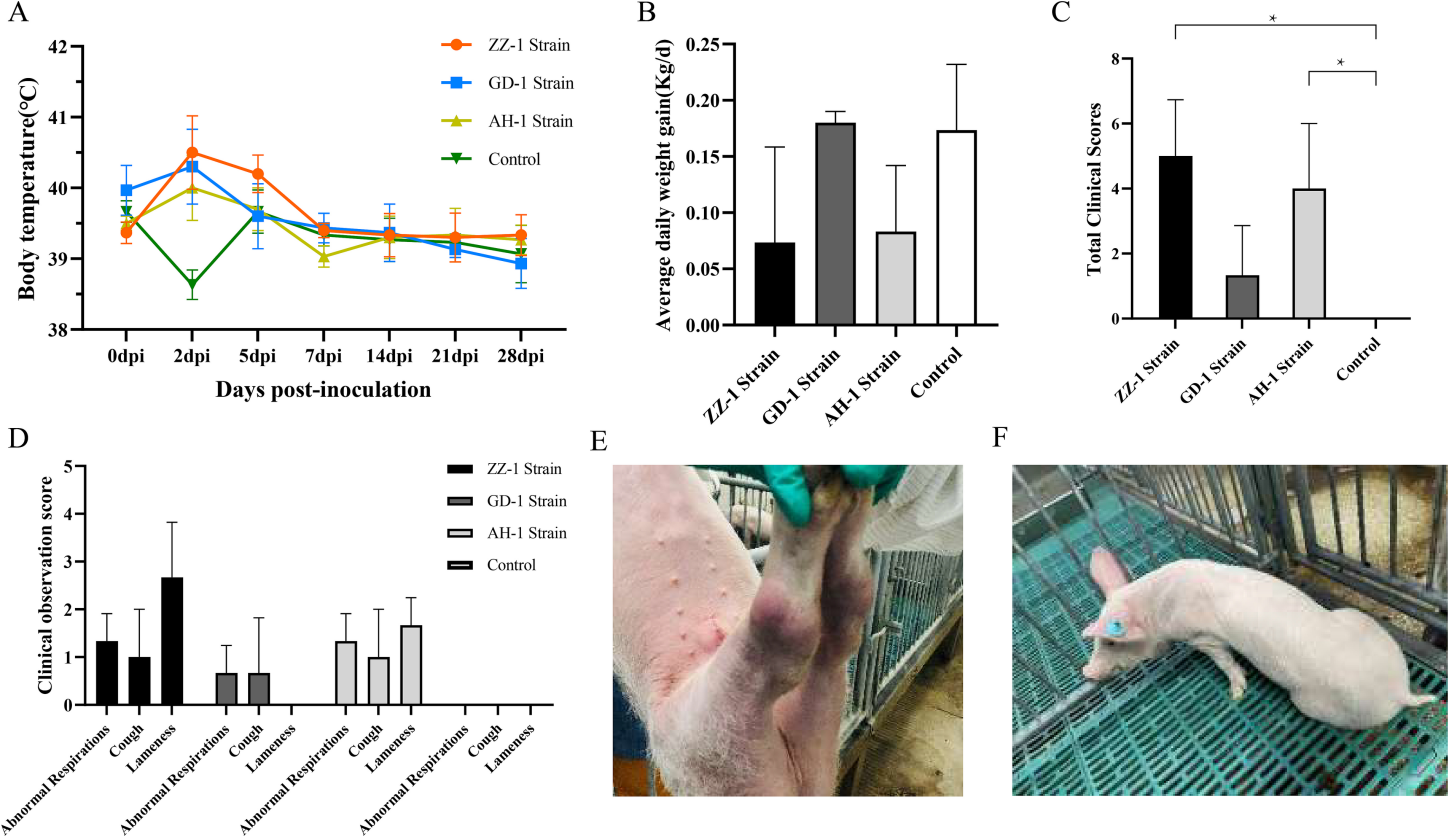
Clinical symptom observation. **(A)** Body temperature of piglets after infection. Temperatures were monitored daily, with peak temperatures at 2 dpi for all infection groups. At 7 dpi, body temperatures in all groups returned to below 40°C. The control group exhibited no significant temperature abnormalities throughout the study. **(B)** Average daily weight gain (ADWG) during the 28-day experimental period. Data are presented as mean ± standard deviation (SD). The ZZ-1 strain and AH-1 strain groups showed lower ADWG than the control group, while the GD-1 strain group displayed no significant difference in ADWG compared to the control group. **(C)** Total clinical observation scores of each group. Data are expressed as mean ± standard deviation (SD). Total scores in ZZ-1 and AH-1 strain groups were significantly increased compared to that in the control group, for which no signs of pathological changes were detected (*p < 0.05). **(D)** Abnormal respiration, cough, and lameness scores of each group. **(E)** Swollen joints observed in the ZZ-1 strain group. **(F)** Persistent and pronounced lameness in the ZZ-1 strain group.

The viable cell counts of three fresh Mhr strains were determined using the CCU method, and piglets were infected with a total dose of 5 × 10^8^ CCU/pig for the ZZ-1 strain and 5 × 10^9^ CCU/pig for the GD-1 and AH-1 strains. Following infection, all three groups exhibited a transient increase in body temperature. The AH-1 strain group peaked at 40.5°C at 2 dpi, the ZZ-1 strain group reached 40.8°C at 2 or 5 dpi, and the GD-1 strain group peaked at 40.7°C at 2 dpi. By 7 dpi, body temperatures in all groups had returned to normal, with no fluctuations exceeding 1.0°C thereafter. In contrast, the control group showed only one piglet reaching 40°C at 5 dpi, with no other temperature abnormalities observed throughout the study (Figure 3A).

During the 28-day experimental period, the ADWG was 0.07 ± 0.0694 kg for the ZZ-1 strain group, 0.18 ± 0.0082 kg for the GD-1 strain group, 0.08 ± 0.0478 kg for the AH-1 strain group, and 0.17 ± 0.0478 kg for the control group. The ADWG in the ZZ-1 and AH-1 strain groups was lower than that of the control group. However, no significant difference in ADWG was observed between the GD-1 strain group and the control group (Figure 3B). Notably, both the ZZ-1 and AH-1 strain groups included one piglet (Z3 and A2, respectively) whose ADWG did not meet expectations. This discrepancy may be attributed to a relatively large initial age, leading to a faster growth rate, or individual differences in pathogen tolerance, resulting in abnormal weight gain compared to group peers. Although the ANOVA *p*-value did not indicate statistical significance (*p*-value > 0.05), effect size analysis revealed a relatively high effect size (*η*^2^ = 0.507), suggesting that group differences account for 50.7% of the total variance.

No clinical signs of *Mycoplasma hyorhinis* infection, such as respiratory distress, coughing, or lameness, were observed in the control group. In contrast, all three infection groups exhibited abnormal respiration, with at least two piglets affected in each group. The ZZ-1 and AH-1 strain groups displayed more severe respiratory symptoms than the GD-1 strain group (Figure 3D). Moderate coughing was observed in one piglet from each infection group (Figure 3D). Coughing began at 14 dpi and persisted until euthanasia in four piglets (Z1, G2, A1, A2).

All piglets in the ZZ-1 and AH-1 strain groups exhibited varying degrees of lameness, with the ZZ-1 strain group displaying higher lameness scores (Figure 3D). Joint swelling, particularly in the tarsal joints, was a common observation (Figure 3E). One piglet (Z1) from the ZZ-1 strain group displayed joint swelling in three limbs, while two piglets (G7, G8) from the GD-1 strain group also exhibited swollen joints. Joint swelling was first detected as early as 5 dpi (Z3, A8) and as late as 21 dpi (Z2). Notably, one piglet (Z3) from the ZZ-1 strain group developed severe lameness, characterized by an inability to stand or walk normally, bearing no weight on both hind limbs, and adopting a dog-sitting posture. This piglet could only walk a few steps with manual assistance, and the lameness remained visibly severe (Figure 3F). No lameness was observed in the GD-1 strain group.

During the entire monitoring period, the ZZ-1 and AH-1 strain groups (5.00 ± 1.41 and 4.00 ± 1.63, respectively) exhibited significantly higher clinical scores than the control group (0.00 ± 0.00, *p* < 0.05) (Figure 3C). The GD-1 strain group (1.33 ± 1.25) did not show a significant difference in clinical scores compared to the control group.

### 3.4 Histopathological observation

**Figure 4:**
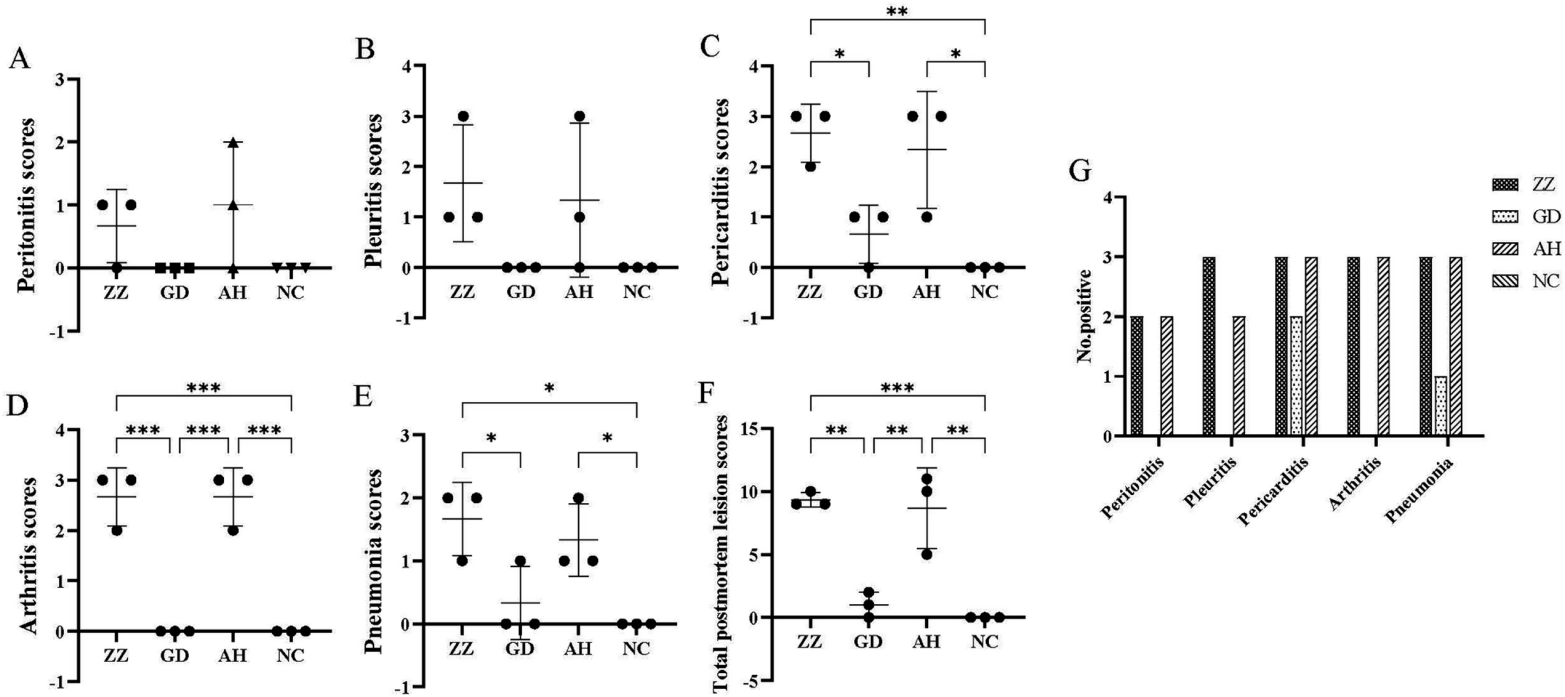
Postmortem observation scores of each group. **(A)** Peritonitis scores of each group. **(B)** Pleuritis scores of each group. **(C)** Pericarditis scores of each group. **(D)** Arthritis scores of each group. **(E)** Pneumonia scores of each group. **(F)** Total Postmortem observation scores of each Group. 9.33 ± 0.47, 1.00 ± 0.82, 8.67 ± 2.62, 0.00 ± 0.00 in ZZ-1, GD-1, AH-1 strain groups and control group, respectively. Data are presented as the mean ± SD. *p < 0.05; **p < 0.01; ***p < 0.001. **(G)** Number of positive animals.

No postmortem signs of disease were observed in the control group. In contrast, pigs in the infection groups exhibited pleuritis, pericarditis, peritonitis, arthritis, and pneumonia, which were documented and scored (Figures 4A–F). The number of animals exhibiting pathological manifestations was quantified (Figure 4G).

**Figure 5:**
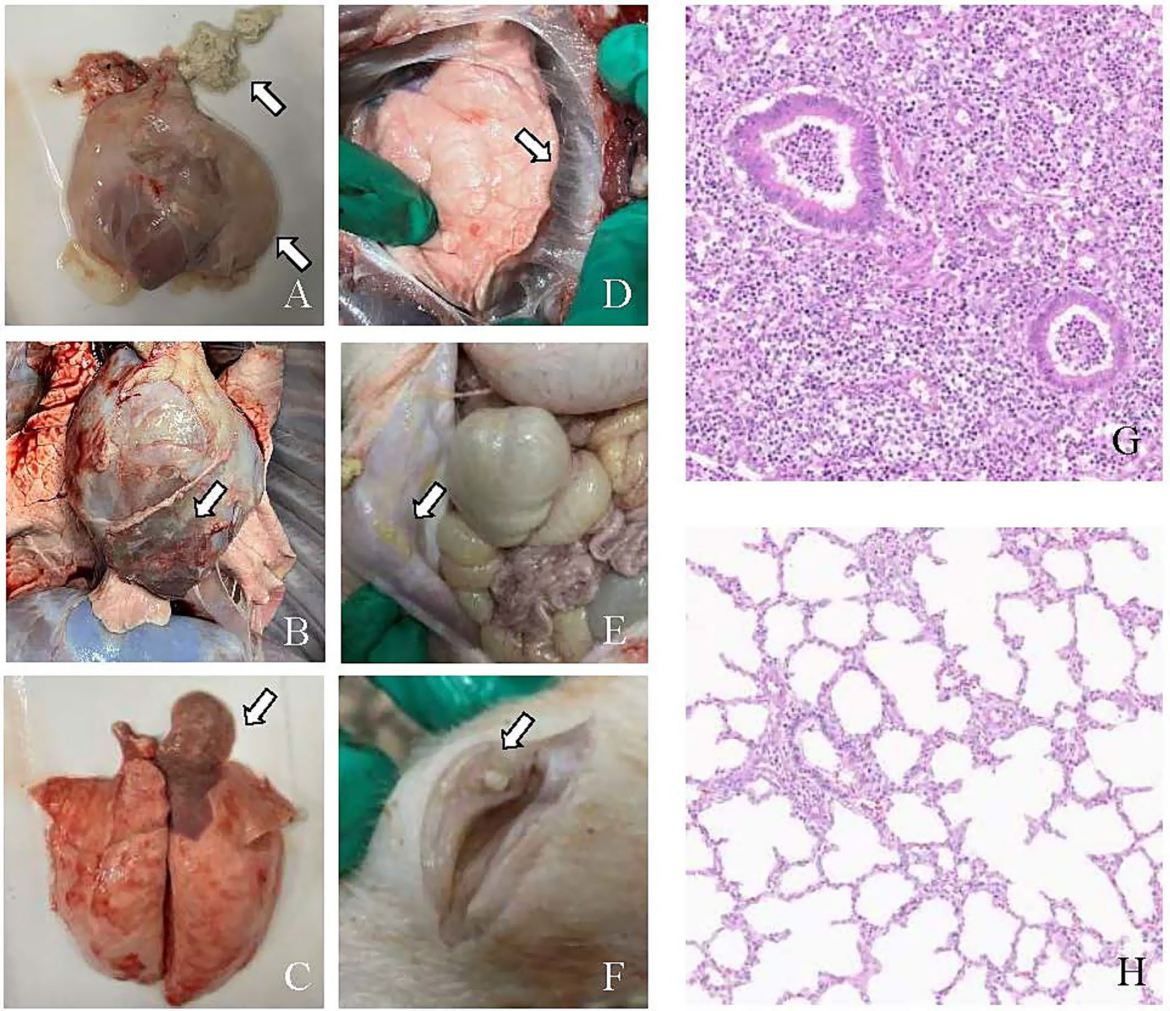
Pathological changes caused by *M. hyorhinis* infection. **(A)** Pericarditis: the arrow indicates the thickening of the pericardium, increased pericardial effusion, and cheese-like exudate between the heart and pericardium. **(B)** Pericarditis: the arrow highlights a close adhesion between the pericardium and the heart. **(C)** Pneumonia: the arrow highlights the shrimp-like lung lesions observed on the dorsal side. **(D)** Pleuritis: the arrow shows fibrinous adhesions between the lung lobes and pleura. **(E)** Peritonitis: the arrow highlighting the cheese-like exudate in the abdominal cavity. **(F)** Arthritis: the arrow points to the abnormal synovial membrane with cheese-like exudate in the articular cavity. **(G)** H&E-stained hepatization-lung tissue of ZZ-1 strain group (Z1), showing obscured or absent alveolar structure, alveolar spaces filled with fibrinous exudates, accompanied by extensive inflammatory cell infiltration and thickened alveolar septa. **(H)** Lung tissue of control group (C11), showing intact alveolar structure, clear alveolar spaces, thin alveolar septa, and minimal inflammatory cell presence.

All pigs in the ZZ-1 strain group exhibited varying degrees of pericarditis and arthritis, ranging from moderate to severe. The pericarditis was characterized by diffuse, severe fibrinous inflammation, pericardial exudate between the heart and pericardium, and significant pericardial thickening. Additionally, increased pericardial effusion was observed (Figures 5A–C). All pigs in this group also displayed moderate to severe arthritis, marked by significant swelling and enlargement of the joint capsule, increased joint fluid, and, in one case (Z2), cheese-like exudate (Figure 5F). One pig (Z1) developed severe pleuritis characterized by diffuse, severe fibrinous pleuritis with thoracic exudate, with most lung lobes adhered to the parietal pleura and interlobar adhesions (Figure 5D). One pig in the ZZ-1 strain group also developed peritonitis, manifesting as a few fibrinous adhesions and yellow cheese-like material in the abdominal cavity (Figure 5E).

In the AH-1 strain group, one pig developed moderate to severe polyserositis (including pleuritis, pericarditis, and peritonitis) and arthritis (A7), another developed moderate pericarditis and severe arthritis (A8), and a third developed severe pericarditis and arthritis (A9). The GD-1 strain group exhibited significantly lower severity scores than the other two infection groups, although increased pericardial fluid accumulation was observed. Notably, pneumonia symptoms were found in at least one pig from each of the three infection groups. Moderate pulmonary consolidation was observed in two pigs from the ZZ-1 strain group (Z1, Z2) and one from the AH-1 strain group (A9). Additionally, pigs Z3, G4, A7, and A8 showed slight consolidation of lung injury.

Histopathological examination of hepatized pig lung tissue, stained with H&E, revealed significant deviations from normal lung architecture. The alveolar spaces were extensively filled with fibrinous exudate, obscuring or destroying the normal structure and creating a solid, liver-like appearance. A pronounced inflammatory response was evident, characterized by heavy infiltration of inflammatory cells, primarily neutrophils and macrophages, within both the alveolar spaces and septa. The alveolar septa were thickened due to interstitial edema, and blood vessels were often dilated and congested. Additionally, extravasated erythrocytes were observed within the alveoli. These findings collectively indicate severe lung tissue inflammation and damage (Figures 5G,H).

Mhr was detected in various tissue samples, including myocardium, pericardial membrane, pericardial fluid, pleural effusion, lung, hilar lymph nodes, tonsil, abdominal effusion, and joint fluid, from the infected groups. The bacteria were successfully re-isolated from all three infection groups (Additional File 4). MLST analysis confirmed that the re-isolated strains had the same sequence types as the original infection strains.

Significant differences in postmortem observations and scores were observed between the three infection groups (*p* < 0.05), indicating varying virulence among the strains. The ZZ-1 strain was the most virulent, consistent with clinical observations.

## 4 Discussion

*Mycoplasma hyorhinis* has a high prevalence in various countries and regions worldwide. In the United States, approximately 40% of sows tested positive for Mhr and *M. hyosynoviae* (36). Additionally, a study found Mhr in up to 97% of 2,710 oral fluid and feces samples (37). Silva et al. examined Mhr in pig tissue samples in the United States from 2017 to 2022, finding a positive rate of 57% (4578/8069), primarily in serosal fibrin and joint samples. Mhr-associated serositis increased annually by 4.2% (38). Similarly, in Austria, Renzhammer et al. analyzed 1,975 samples collected between 2016 and 2021, with Mhr having the highest detection rate, reaching 55.1% (39).

Generally, the samples analyzed primarily originate from upper respiratory tract swabs, joint fluid, and serous membranes, while researchers often prioritize examining lung tissues for *M. hyopneumoniae*. In this study, the positivity rate of Mhr was 31.77% among 1,001 lung samples collected from China in 2022. Additionally, the study observed that the positivity rate of Mhr was highest in April, May, and August (63.75, 40.18, and 39.36%, respectively), while rates were lower in January, February, March, and November (17.65, 29.55, 14.15, and 18.75%, respectively). A province-based analysis revealed that Jiangsu had the highest positivity rate of Mhr (40.00%), followed by Hubei at 36.73%. These findings suggest a correlation between the positivity rate of Mhr and both seasonal factors (or temperature) and geographical regions.

Currently, the full extent of Mhr’s pathogenic potential remains uncertain. Some studies that used intranasal or tonsillar inoculation did not observe clinical symptoms or visible lesions (40, 41). This suggests that a single-dose intranasal inoculation may not be sufficient to induce all typical lesions, and multiple inoculation routes may be more suitable for establishing a challenge model (4, 26, 42–44). This study established piglet infection models by inoculating them via multiple routes and assessed clinical symptoms and histopathological changes. All three strains used in the virulence study were isolated from lung tissue samples. We observed that piglets infected with Mhr experienced transient fever, and piglets infected with highly virulent strains showed a trend toward reduced ADWG, though the difference was not statistically significant. Additionally, some piglets displayed joint swelling, with severe cases leading to lameness. These clinical observations (fever, weight loss, and lameness) are consistent with previous studies (43, 45). The ZZ-1 and AH-1 strains induced typical and extensive symptoms in piglets, including pleuritis, peritonitis, pericarditis, arthritis, and pneumonia, which aligns with findings from Földi D and Martinson B et al. (26, 45). The pericarditis observed in piglets in our study was mainly serous and fibrinous, which aligns with the findings of Földi et al. (45). However, naturally infected piglets at a similar age typically show fibrinous pericarditis (5). This discrepancy may be due to differences in the age at infection or strain virulence. Given the limited number of reports, it is difficult to conclude whether pericarditis induced by coinfection and natural infection follows a consistent pattern. Notably, the three Mhr strains exhibited varying levels of virulence. Based on clinical observations and necropsy results, the ZZ-1 strain demonstrated the highest virulence, while the GD-1 strain exhibited the lowest, offering valuable insights into the pathogenicity of different Mhr strains and their impact on swine health. This study lays the groundwork for a database of high- and low-virulence strains, essential for virulence target screening and precise diagnostic development. Highly virulent strains may serve as ideal candidates for vaccine development, enabling the selection of effective and broadly protective vaccines, while also providing a basis for evaluating vaccine efficacy.

Moving forward, it is imperative to intensify our focus on Mhr. Strengthening the study of its pathogenicity and expanding the scope of epidemiological investigations is essential. This study, being a smaller-scale “pilot study,” aims to provide preliminary insights into the pathogenicity and virulence of Mhr strains, which will guide the design of larger, more comprehensive studies in the future. Additionally, establishing infection models with a broader range of strains and animals will deepen our understanding of its pathogenic mechanisms. Investigating the genetic differences between various strains from a molecular biology perspective will shed light on the underlying causes of these differences. Developing methods for the rapid differentiation of highly virulent and less virulent strains, coupled with advancing vaccine research, will be crucial for the effective prevention and control of Mhr-related diseases.

## 5 Conclusion

Overall, this study assessed the positive rate of Mhr in clinical lung samples from pigs, successfully isolated three wild strains of Mhr, and established infection models to evaluate their virulence. The results indicated that Mhr can cause multiple serositis, pneumonia, and arthritis in piglets, with notable differences in virulence among the various strains.

## Data Availability

The original contributions presented in the study are included in the article/supplementary material, further inquiries can be directed to the corresponding author.

## Supplementary material

ADDITIONAL FILE 1 Oligonucleotide primers for amplification and sequencing of *Mycoplasma hyorhinis* MLST.

ADDITIONAL FILE 2 Description of clinical observation scoring.

ADDITIONAL FILE 3 Postmortem observations and scores of relative lesion severity.

ADDITIONAL FILE 4 Detection of *M. hyorhinis* in postmortem tissues from virulence experiments.

## References

[1] Siqueira FM Thompson CE Virginio VG Gonchoroski T Reolon L Almeida LG. New insights on the biology of swine respiratory tract mycoplasmas from a comparative genome analysis. BMC Genomics. (2013) 14:175. doi: 10.1186/1471-2164-14-175 23497205 PMC3610235

[2] Ferrarini MG Siqueira FM Mucha SG Palama TL Jobard É Elena-Herrmann B. Insights on the virulence of swine respiratory tract mycoplasmas through genome-scale metabolic modeling. BMC Genomics. (2016) 17:353. doi: 10.1186/s12864-016-2644-z 27178561 PMC4866288

[3] Decker JL Barden JA. *Mycoplasma hyorhinis* swine arthritis. Arthritis Rheum. (1971) 14:781. doi: 10.1002/art.1780140617 PMID: 5135797

[4] Lin JH Chen SP Yeh KS Weng CN. *Mycoplasma hyorhinis* in Taiwan: diagnosis and isolation of swine pneumonia pathogen. Vet Microbiol. (2006) 115:111–6. doi: 10.1016/j.vetmic.2006.02.004 PMID: 16540266

[5] Ustulin M Rossi E Vio D. A case of pericarditis caused by *Mycoplasma hyorhinis* in a weaned piglet. Porcine Health Manag. (2021) 7:32. doi: 10.1186/s40813-021-00211-4 33845919 PMC8040207

[6] Friis NF Feenstra AA. *Mycoplasma hyorhinis* in the etiology of serositis among piglets. Acta Vet Scand. (1994) 35:93–8. doi: 10.1186/BF03548359 PMID: 8209825 PMC8101397

[7] Jansson E Backman A Hakkarainen K Miettinen A Seniusová B. Mycoplasmas and arthritis. Z Rheumatol. (1983) 42:315–9. PMID: 6421028

[8] Resende TP Pieters M Vannucci FA. Swine conjunctivitis outbreaks associated with *Mycoplasma hyorhinis*. J Vet Diagnostic Investig. (2019) 31:766–9. doi: 10.1177/1040638719865767, PMID: 31342882 PMC6727109

[9] Friis NF. Mycoplasms of the swine--a review. Nord Vet Med. (1975) 27:329–36. PMID: 1153279

[10] Friis NF Kokotovic B Svensmark B. *Mycoplasma hyorhinis* isolation from cases of otitis media in piglets. Acta Vet Scand. (2002) 43:191–3. PMID: 12564549

[11] Morita T Sasaki A Kaji N Shimada A Kazama S Yagihashi T. Induction of temporary otitis media in specific-pathogen-free pigs by intratympanic inoculation of *Mycoplasma hyorhinis*. Am J Vet Res. (1998) 59:869–73. PMID: 9659554

[12] Bünger M Brunthaler R Unterweger C Loncaric I Dippel M Ruczizka U. *Mycoplasma hyorhinis* as a possible cause of fibrinopurulent meningitis in pigs? - a case series. Porcine Health Manag. (2020) 6:38. doi: 10.1186/s40813-020-00178-8, PMID: 33292668 PMC7713030

[13] Ko CC Merodio MM Spronk E Lehman JR Shen H Li G. Diagnostic investigation of *Mycoplasma hyorhinis* as a potential pathogen associated with neurological clinical signs and central nervous system lesions in pigs. Microb Pathog. (2023) 180:106172. doi: 10.1016/j.micpath.2023.106172 37230257

[14] Suk RN Helke KL Fitzgerald DC Hassid M McVadon D Taylor CL. Bacteria endocarditis caused by *Mycoplasma hyorhinis* in a juvenile, immunosuppressed pig (*Sus scrofa domesticus*) following partial heart transplantation. Comp Med. (2024) 74:295–303. doi: 10.30802/AALAS-CM-23-000090, PMID: 38749668 PMC11373685

[15] Lee JA Oh YR Hwang MA Lee JB Park SY Song CS. *Mycoplasma hyorhinis* is a potential pathogen of porcine respiratory disease complex that aggravates pneumonia caused by porcine reproductive and respiratory syndrome virus. Vet Immunol Immunopathol. (2016) 177:48–51. doi: 10.1016/j.vetimm.2016.06.008, PMID: 27436444

[16] Thakor JC Sahoo M Karam Pal S Rajendra S Salauddin Q Ajay K. Porcine respiratory disease complex (PRDC) in Indian pigs: a slaughterhouse survey. Vet Ital. (2023) 59:23–38. doi: 10.12834/VetIt.2935.20591.2, PMID: 37994635

[17] Clavijo MJ Murray D Oliveira S Rovira A. Infection dynamics of *Mycoplasma hyorhinis* in three commercial pig populations. Vet Rec. (2017) 181:68. doi: 10.1136/vr.104064, PMID: 28424318

[18] Luehrs A Siegenthaler S Grützner N Grosse Beilage E Kuhnert P Nathues H. Occurrence of *Mycoplasma hyorhinis* infections in fattening pigs and association with clinical signs and pathological lesions of enzootic pneumonia. Vet Microbiol. (2017) 203:1–5. doi: 10.1016/j.vetmic.2017.02.001, PMID: 28619130

[19] Fourour S Fablet C Tocqueville V Dorenlor V Eono F Eveno E. A new multiplex real-time TaqMan(®) PCR for quantification of *Mycoplasma hyopneumoniae*, *M. hyorhinis* and *M. flocculare*: exploratory epidemiological investigations to research mycoplasmal association in enzootic pneumonia-like lesions in slaughtered pigs. J Appl Microbiol. (2018) 125:345–55. doi: 10.1111/jam.13770, PMID: 29603531

[20] Lobo E Poveda C Gupta R Suarez A Hernández Y Ramírez A. Mycoplasmas hyorhinis in different regions of Cuba diagnosis. Braz J Microbiol. (2011) 42:721–5. doi: 10.1590/S1517-838220110002000039 24031686 PMC3769822

[21] L’Ecuyer C Boulanger P. Enzootic pneumonia in pigs: identification of a causative mycoplasma in infected pigs and in cultures by immunofluorescent staining. Can J Comp Med. (1970) 34:38–46. 4246002 PMC1319418

[22] Wang J Hua L Gan Y Yuan T Li L Yu Y. Virulence and inoculation route influence the consequences of *Mycoplasma hyorhinis* infection in Bama miniature pigs. Microbiol Spectr. (2022) 10:e0249321. doi: 10.1128/spectrum.02493-21, PMID: 35446115 PMC9241778

[23] Yamaguti M Oliveira RC Marques LM Buzinhani M Buim MR Neto RL. Molecular characterisation of *Mycoplasma hyorhinis* isolated from pigs using pulsed-field gel electrophoresis and 16S rRNA sequencing. Vet Record Open. (2015) 2:e000093. doi: 10.1136/vetreco-2014-000093, PMID: 26688737 PMC4680736

[24] Bekő K Felde O Sulyok KM Kreizinger Z Hrivnák V Kiss K. Antibiotic susceptibility profiles of *Mycoplasma hyorhinis* strains isolated from swine in Hungary. Vet Microbiol. (2019) 228:196–201. doi: 10.1016/j.vetmic.2018.11.027, PMID: 30593367

[25] Martinson B Minion FC Kroll J Hermann J. Age susceptibility of caesarian derived colostrum deprived pigs to *Mycoplasma hyorhinis* challenge. Vet Microbiol. (2017) 210:147–52. doi: 10.1016/j.vetmic.2017.09.005, PMID: 29103684

[26] Martinson B Zoghby W Barrett K Bryson L Christmas R Minion FC. Efficacy of an inactivated *Mycoplasma hyorhinis* vaccine in pigs. Vaccine. (2018) 36:408–12. doi: 10.1016/j.vaccine.2017.11.063, PMID: 29221894

[27] Gois M Pospisil Z Cerny M Mrva V. Production of pneumonia after intransal inoculation of gnotobiotic piglets with three strains of *Mycoplasma hyorhinis*. J Comp Pathol. (1971) 81:401–10. doi: 10.1016/0021-9975(71)90028-4 PMID: 4935555

[28] Zhang Y Gan Y Wang J Feng Z Zhong Z Bao H. Dysbiosis of gut microbiota and intestinal barrier dysfunction in pigs with pulmonary inflammation induced by *Mycoplasma hyorhinis* infection. mSystems. (2022) 7:e0028222. doi: 10.1128/msystems.00282-22, PMID: 35699454 PMC9426446

[29] Wang J Yu Y Li Y Li S Wang L Wei Y. A multifunctional enolase mediates cytoadhesion and interaction with host plasminogen and fibronectin in *Mycoplasma hyorhinis*. Vet Res. (2022) 53:26. doi: 10.1186/s13567-022-01041-0, PMID: 35337383 PMC8951703

[30] Wang J Li Y Pan L Li J Yu Y Liu B. Glyceraldehyde-3-phosphate dehydrogenase (GAPDH) moonlights as an adhesin in *Mycoplasma hyorhinis* adhesion to epithelial cells as well as a plasminogen receptor mediating extracellular matrix degradation. Vet Res. (2021) 52:80. doi: 10.1186/s13567-021-00952-8, PMID: 34082810 PMC8173509

[31] Palzer A Ritzmann M Wolf G Heinritzi K. Associations between pathogens in healthy pigs and pigs with pneumonia. Vet Rec. (2008) 162:267–71. doi: 10.1136/vr.162.9.267 PMID: 18310558

[32] Wang J Gan Y Yuan T Huang Y Zhang L Wei Y. Protection against *Mycoplasma hyorhinis* infection in commercial pigs via immunization with inactivated vaccines prepared with homologous or heterologous strains. Vaccine. (2024) 42:126421. doi: 10.1016/j.vaccine.2024.126421, PMID: 39388932

[33] Jolley KA Bray JE Maiden MCJ. Open-access bacterial population genomics: BIGSdb software, the PubMLST.org website and their applications. Wellcome Open Res. (2018) 3:124. doi: 10.12688/wellcomeopenres.14826.1 30345391 PMC6192448

[34] Trüeb B Catelli E Luehrs A Nathues H Kuhnert P. Genetic variability and limited clonality of *Mycoplasma hyorhinis* in pig herds. Vet Microbiol. (2016) 191:9–14. doi: 10.1016/j.vetmic.2016.05.015, PMID: 27374901

[35] Garcia-Morante B Segalés J Fraile L Pérez de Rozas A Maiti H Coll T. Assessment of *Mycoplasma hyopneumoniae*-induced pneumonia using different lung lesion scoring systems: a comparative review. J Comp Pathol. (2016) 154:125–34. doi: 10.1016/j.jcpa.2015.11.003, PMID: 26774274

[36] Roos LR Surendran Nair M Rendahl AK Pieters M. Mycoplasma hyorhinis and *Mycoplasma hyosynoviae* dual detection patterns in dams and piglets. PLoS One. (2019) 14:e0209975. doi: 10.1371/journal.pone.0209975, PMID: 30605453 PMC6317828

[37] Gerszon J Büchse A Genz B Pollock Y Gleeson B Morris A. The use of oral fluids and sock samples for monitoring key pathogens in pig populations for surveillance purposes. Prev Vet Med. (2024) 228:106237 38820832 10.1016/j.prevetmed.2024.106237

[38] Silva A Almeida M Michael A Rahe MC Siepker C Magstadt DR. Detection and disease diagnosis trends (2017-2022) for *Streptococcus suis*, *Glaesserella parasuis*, *Mycoplasma hyorhinis*, *Actinobacillus suis*, and *Mycoplasma hyosynoviae* at Iowa State University veterinary diagnostic laboratory. BMC Vet Res. (2023) 19:268. doi: 10.1186/s12917-023-03807-w, PMID: 38087358 PMC10714645

[39] Renzhammer R Auer A Loncaric I Entenfellner A Dimmel K Walk K. Retrospective analysis of the detection of pathogens associated with the porcine respiratory disease complex in routine diagnostic samples from Austrian swine stocks. Vet Sci. (2023) 10:601. doi: 10.3390/vetsci10100601 PMC10610783 37888553

[40] Merodio M McDaniel A Poonsuk K Magtoto R Ferreyra FSM Meiroz-De-Souza-Almeida H. Evaluation of colonization, variable lipoprotein-based serological response, and cellular immune response of *Mycoplasma hyorhinis* in experimentally infected swine. Vet Microbiol. (2021) 260:109162 doi: 10.1016/j.vetmic.2021.109162 34217902

[41] Gomes Neto JC Strait EL Raymond M Ramirez A Minion FC. Antibody responses of swine following infection with *Mycoplasma hyopneumoniae*, *M. hyorhinis*, *M. hyosynoviae*, and *M. flocculare*. Vet Microbiol. (2014) 174:163–71. doi: 10.1016/j.vetmic.2014.08.008, PMID: 25240775

[42] Barden JA Decker JL Dalgard DW Aptekar RG. *Mycoplasma hyorhinis* swine arthritis. 3. Modified disease in piney woods swine. Infect Immun. (1973) 8:887–90. doi: 10.1128/iai.8.6.887-890.1973 PMID: 4784887 PMC422945

[43] Chen D Wei Y Huang L Wang Y Sun J Du W. Synergistic pathogenicity in sequential coinfection with Mycoplasma hyorhinis and porcine circovirus type 2. Vet Microbiol. (2016) 182:123–30. doi: 10.1016/j.vetmic.2015.11.003, PMID: 26711038

[44] Gois M Kuksa F Sisák F. Experimental infection of gnotobiotic piglets with Mycoplasma hyorhinis and *Bordetella bronchiseptica*. Zentralblatt fur Veterinarmedizin Reihe B J Vet Med Ser B. (1977) 24:89–96. doi: 10.1111/j.1439-0450.1977.tb00978.x PMID: 842196

[45] Földi D Nagy ZE Belecz N Szeredi L Földi J Kollár A. Establishment of a *Mycoplasma hyorhinis* challenge model in 5-week-old piglets. Front Microbiol. (2023) 14:1209119. doi: 10.3389/fmicb.2023.1209119 37601388 PMC10436309

