# Supplementary material for: Isolation and identification of Mycoplasma hyorhinis and virulence evaluation of its field isolates
Source: Front Vet Sci. 2025 Jun 18;12:1542992. doi: 10.3389/fvets.2025.1542992 (PMC12213342; doi:10.3389/fvets.2025.1542992)
Supplement: ADDITIONAL FILE 1 — Oligonucleotide primers for amplification and sequencing of Mycoplasma hyorhinis MLST. [file Table_2.docx]

Supplementary Material

**Supplementary Table 1**

Oligonucleotide Primers for Amplification and Sequencing of *Mycoplasma hyorhinis* MLST

| Locus | Forward(5` to 3`) | reverse(5` to 3`) | Amplicon  size (bp) |
| --- | --- | --- | --- |
| dnaA | CCAGAAGTCTTAGGTGGTTTTGA | ATGATCCTTGCCTCCAAAAA | 495 |
| rpoB | CAACGTCAAGCTGTTCCATT | CCTGCACTAACTTCTGATCCAA | 563 |
| gyrB | CCGATTCTGATGGTTCACAT | TTTTTGCATATTTTGCATTTTCTT | 399 |
| gltX | GCTGAAAGACTCTCAAAATCACC | CAAGCCTTTTTGAAATTAGTTCTTT | 493 |
| adk | GCGATGGCATCTAATTCTTTT | ACTCAGGCAAAGTTTTTAGAACA | 484 |
| gmk | TTGCGCCTGTTTCTGTTAAT | AAGAGACAAAAGACCTAATGAAGTAGA | 499 |

**Supplementary Table 4**

Detection of *M. hyorhinis* in Postmortem Tissues from Virulence Experiments

| tissue | ZZ-Group | GD-Group | AH-Group | Control Group |
| --- | --- | --- | --- | --- |
| Myocardium | 0/3 | 0/3 | 0/3 | 0/3 |
| Pricardial membrane | 3/3 | 0/3 | 2/3 | 0/3 |
| Pricardial fluid | 3/3 | 1/3 | 2/3 | 0/3 |
| Peural effusion | 3/3 | 0/3 | 3/3 | 0/3 |
| Lung | 3/3 | 2/3 | 3/3 | 0/3 |
| Hilar lymph nodes | 3/3 | 0/3 | 2/3 | 0/3 |
| Tonsil | 3/3 | 3/3 | 3/3 | 0/3 |
| Abdominal effusion | 2/3 | 0/3 | 2/3 | 0/3 |
| Joint fluid | 3/3 | 0/3 | 3/3 | 0/3 |

**Supplementary Table 2**

Description of Clinical Observation Scoring

| Score | Abnormal Respirations | Cough | Lameness |
| --- | --- | --- | --- |
| 0 | **Normal**—no respiratory discomfort | **Normal**—no cough | **Normal**—no visible lameness at a walk |
| 1 | **Mild**—mild increase in respiratory rate | **Mild**—slight cough that does not seem to disturb normal activities | **Mild**—difficult to observe lameness as the animal walks around the pen; not constantly lame when walking; walks at a normal speed; is weight bearing while walking and standing. Lameness is indicated by intermittent reduced weight bearing on one limb or shortening of the stride. |
| 2 | **Moderate**—notable increase in respiratory rate | **Moderate**—loud, pronounced cough that disrupts normal activities | **Moderate**—constantand observed throughout every step at a walking pace; bearing some weight on the leg at a walk and standing but short-striding one or more legs while walking; walks at a normal speed. Animals may appear to stand hunched with limbs extended farther cranially than expected in an attempt to shift weight to the pelvic limbs. |
| 3 | **Severe**—thumping | **Severe**—dry, hacking cough that appears painful | **Severe**-puts no weight on the leg(s) the first few steps after standing; constantobvious lameness while at a walking pace; putting very little to no weight on the leg(s) at a walk or while standing. Lameness requires the pig to slow its speed of walking. |
| 4 | n/a | n/a | **Recumbent**—will not stand even with assistance. |

**Supplementary Table 3**

Postmortem Observations and Scores of Relative Lesion Severity

| Disease Sign | Assessment factors | Score |
| --- | --- | --- |
| Peritonitis | No signs of peritonitis | 0 |
|  | Few fibrinous adhesions | 1 |
|  | Diffuse, moderate fibrinous peritonitis | 2 |
|  | Diffuse severe fibrinous or fibrino-purulent peritonitis | 3 |
| Pleuritis | No signs of pleuritis | 0 |
|  | Few focal fibrinous adhesions on the visceral or parietal pleura | 1 |
|  | Diffuse moderate fibrinous pleuritis | 2 |
|  | Diffuse severe fibrinous or fibrino-purulent pleuritis with thoracic exudate | 3 |
| Pericarditis | No signs of pericarditis | 0 |
|  | Few focal fibrinous pericardial adhesions | 1 |
|  | Diffuse moderate fibrinous pericarditis | 2 |
|  | Diffuse severe fibrinous or fibrino-purulent pericarditis with pericardial exudate | 3 |
| Arthritis | No signs of arthritis | 0 |
|  | Slight or moderate hyperaemia or edema of joint capsule with moderate increase of normal synovial fluid in one or two joints | 1 |
|  | Moderately increased turbid synovial fluid and moderate, edematous villous hypertrophy in one or more joints | 2 |
|  | Highly increased very turbid synovial fluid and very edematous villous hypertrophy in one or more joints | 3 |
| Pneumonia | No signs of pneumonia | 0 |
|  | Slight consolidation of lung injury | 1 |
|  | Moderate consolidation of lung injury | 2 |
|  | Severe consolidation of lung injury | 3 |
