# Supplementary material for: Isolation and identification of Mycoplasma hyorhinis and virulence evaluation of its field isolates
Source: Front Vet Sci. 2025 Jun 18;12:1542992. doi: 10.3389/fvets.2025.1542992 (PMC12213342; doi:10.3389/fvets.2025.1542992)

Original Images

Figure 2 Identification of *M. hyorhinis* isolates

Figure 2 A-a

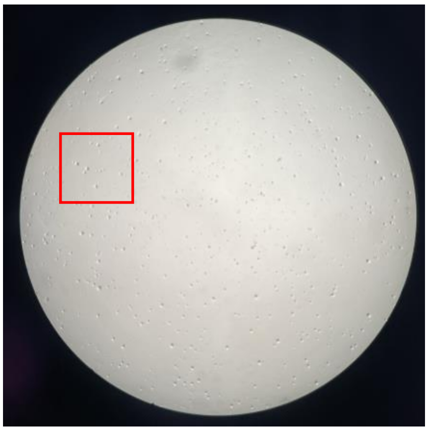

Figure 2 A-b

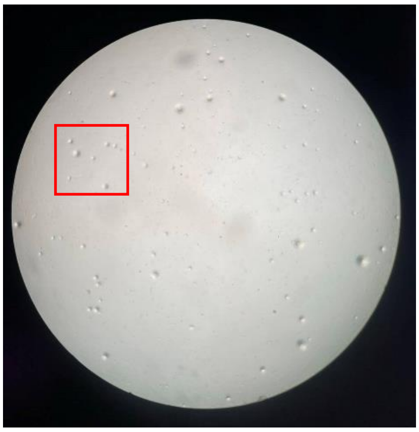

Figure 2 A-c

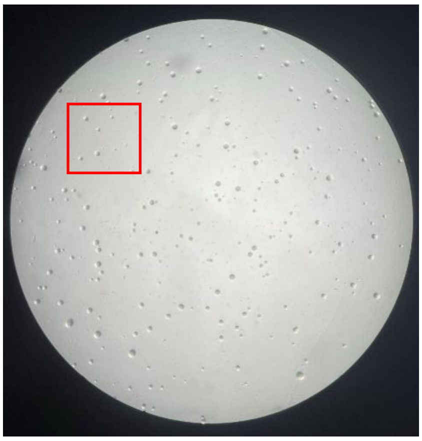

Figure 2 B

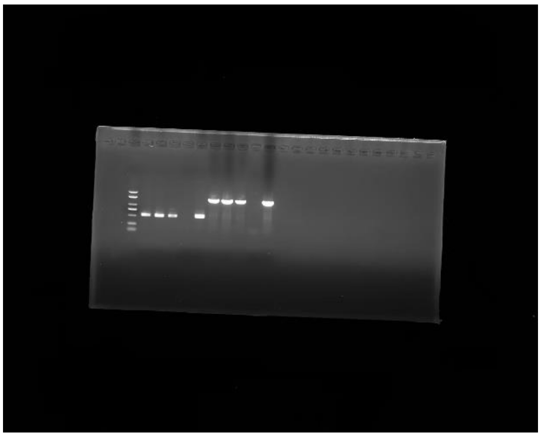

Supplement: Supplementary file 3 [file Image_1.pdf]
